# New Care Models revisited

**DOI:** 10.1080/17571472.2017.1299163

**Published:** 2017-03-08

**Authors:** Paul Thomas

This Issue of *LJPC* (9.2) is deliberately short, to complement a Virtual Issue on New Care Models, also presented in March 2017. The Virtual Issue highlights 18 previously published *LJPC* papers that have explored different aspects of integrated care and health promotion at local, community level. These papers will support discussions at the 30 March 2017 London City Health Conference, hosted by RCGP London in collaboration with *LJPC*. The City Health Conference focuses on New Models of Care – ways to integrate care and health promotion at local, community level.

Simply put, general practice cannot achieve on its own everything that general practice needs to achieve. Its traditional values of whole person, community-oriented care and health promotion need to be embedded within whole communities, networks and systems. This means systems to support a grand alliance for health and care – horizontally for inter-disciplinary team-working that builds communities for health, and vertically for generalist/specialist collaboration for good medical care. The question is, *how we do this in sustainable and efficient ways*?

The two papers in Issue 9.2 build from the 18 papers in the Virtual Issue on New Care Models

- Lee and Titchener describe the GSTT@home service. This initiative brings specialist care into patients homes to treat relapse of chronic conditions, infections requiring IV therapy, heart failure, urinary tract infections, falls, community acquired pneumonia, diabetes and blood monitoring. It provides a transferable model of complex collaborations for the care of sick people at home.
- Kelley-Patterson, Knapton and Hurst describe ways to generate comparative data from large databases to demonstrate the effect of complex collaborations such as hospital at home, to scrutinise and defend variation, and to take ownership of decision-making at a local level. It would be possible to use such data in annual cycles of learning and change to see the combined effect of multiple initiatives for health and care.

*LJPC* welcomes papers that help to understand how to practically achieve integrated care and integrated health promotion at local, community level. Please keep them coming.

